# Why being an expert – despite xpert –remains crucial for children in high TB burden settings

**DOI:** 10.1186/s12879-017-2236-9

**Published:** 2017-02-06

**Authors:** Jason M. Bacha, Katherine Ngo, Petra Clowes, Heather R. Draper, Elias N. Ntinginya, Andrew DiNardo, Chacha Mangu, Issa Sabi, Bariki Mtafya, Anna M. Mandalakas

**Affiliations:** 1 Baylor College of Medicine Children’s Foundation – Tanzania, Centre of Excellence at Mbeya Zonal Referral Hospital, Mbeya, Tanzania; 2 Baylor International Pediatric AIDS Initiative (BIPAI) at Texas Children’s Hospital, Baylor College of Medicine, Houston, TX USA; 3 The Global Tuberculosis Program, Texas Children’s Hospital, Global and Immigrant Health, Department of Pediatrics Baylor College of Medicine, Houston, TX 77030 USA; 4 National Institute of Medical Research–Mbeya Medical Research Centre, Mbeya, Tanzania

**Keywords:** Childhood TB, Clinical diagnosis, Diagnostic test performance, Tanzania

## Abstract

**Background:**

As access to Xpert expands in high TB-burden settings, its performance against clinically diagnosed TB as a reference standard provides important insight as the majority of childhood TB is bacteriologically unconfirmed. We aim to describe the characteristics and outcomes of children with presumptive TB and TB disease, and assess performance of Xpert under programmatic conditions against a clinical diagnosis of TB as a reference standard.

**Methods:**

Retrospective review of children evaluated for presumptive TB in Mbeya, Tanzania. Baseline characteristics were compared by TB disease status and, for patients diagnosed with TB, by TB confirmation status using Wilcoxon rank sum test for continuous variables and the Chi-square test for categorical variables. Sensitivity and specificity were calculated to assess the performance of Xpert, smear, and culture against clinical TB. Kappa statistics were calculated to assess agreement between Xpert and smear to culture.

**Results:**

Among children (N = 455) evaluated for presumptive TB, 70.3% (320/455) had Xpert and 62.8% (286/455) had culture performed on sputa. 34.5% (157/455) were diagnosed with TB: 80.3% (126/157) pulmonary TB, 13.4% (21/157) bacteriologically confirmed, 53.5% (84/157) HIV positive, and 48.4% (76/157) inpatients. Compared to the reference standard of clinical diagnosis, sensitivity of Xpert was 8% (95% CI 4–15), smear 6% (95% CI 3–12) and culture 16% (95% CI 9–24), and did not differ based on patient disposition, nutrition or HIV status.

**Conclusion:**

Despite access to Xpert, the majority of children with presumptive TB were treated based on clinical diagnosis. Reflecting the reality of clinical practice in resource limited settings, new diagnostics such as Xpert serve as important adjunctive tests but will not obviate the need for astute clinicians and comprehensive diagnostic algorithms.

**Electronic supplementary material:**

The online version of this article (doi:10.1186/s12879-017-2236-9) contains supplementary material, which is available to authorized users.

## Background

In 2014, one million cases of childhood tuberculosis (TB) resulted in 140,000 pediatric deaths worldwide [1]. Difficulties in diagnosing TB in children, coupled with the risk of rapid, life-threatening progression of untreated disease, make childhood TB a public health crisis in high TB burden settings. Global data suggest that only 35% of pediatric cases are diagnosed and notified [2]. The true magnitude of childhood TB in high burden settings is difficult to ascertain due to under-diagnosis and under-reporting [3, 4]. Moreover in Tanzania only 23% of an estimated 270,000 TB cases were notified in 2014, and children comprised just 10% of these notified cases [1].

Firm diagnosis and confirmation of childhood TB remain challenging due to bacteriologic confirmation by culture, the universally acknowledged reference standard, being suboptimal in children. In children, the sensitivity of culture varies (range 1.5%–65%) depending on age, disease severity, number of specimens collected and disease prevalence of the setting [5–8]. The introduction of the Xpert MTB/RIF assay (Xpert; Cepheid, Sunnyvale, CA, USA) brought promise as it showed improved sensitivity, specificity and accuracy compared to smear microscopy in culture-positive pediatric cases [5, 9, 10]. Xpert was endorsed by World Health Organization (WHO) as an initial diagnostic test in children, though as a conditional recommendation with very low quality of evidence [11]. However, when evaluated in respiratory samples from culture-negative children started on anti-tuberculosis therapy, Xpert sensitivity was only 2–4% [5, 11]. Due to the paucibacilllary nature of the disease in children, the majority of childhood TB cases are smear and culture negative, highlighting a potential limitation when applying Xpert in these cases.

Subsequently, even with access to Xpert, the diagnosis of childhood TB in high TB-burden countries such as Tanzania relies upon the clinician’s ability to synthesize symptoms, radiographic findings, TB exposure history, and/or immunologic evidence of *Mycobacterium tuberculosis* infection. The use of a clinical TB diagnosis as a reference standard against which to evaluate the performance of diagnostic tests is an imperfect approach, but it affords an important perspective on test performance in this setting. As Tanzania and other high TB-burden countries roll out Xpert technology across expanding levels of care, its performance in programmatic conditions must be continually evaluated to effectively inform policy makers. Upfront Xpert testing in children has shown superior yield over smear microscopy [12, 13], but further assessment in children presenting with a wide variety of disease manifestations in varied settings is needed. In our retrospective descriptive study, we described the characteristics and outcomes of children with presumptive TB and TB disease, and assessed the performance of Xpert under programmatic conditions against a clinical diagnosis of TB as a reference standard. The approach was applied among children with a range of disease severity in the context of an outpatient pediatric clinic in Mbeya, Tanzania.

## Methods

### Ethical approval

Approval was obtained from all necessary ethical bodies including the Mbeya Medical Research and Ethics Committee and the National Institute of Medical Research (NIMR) in Tanzania, and the Institutional Review Board, Baylor College of Medicine, Houston, Texas, USA. Waiver of consent was approved by all committees as this retrospective study analyzed only de-identified data.

### Study setting

This retrospective descriptive study was conducted at the Baylor College of Medicine Children’s Foundation – Tanzania Centre of Excellence (COE) at Mbeya Zonal Referral Hospital (MZRH), the single zonal referral hospital for the Southern Highlands Zone of Tanzania, with a catchment area serving over 3.2 million children aged 0–14 years old [14]. The Southern Highlands Zone reported more than 10% of Tanzania’s notified TB cases [15]. The estimated burden of multidrug-resistant TB in Tanzania is <1% [1]. The Mbeya COE is a family-centered, pediatric prevention, care and treatment centre offering comprehensive care to both HIV positive and HIV negative children affected by TB, malnutrition and other chronic conditions. A variety of different cadres, including medical officers, nurses and paediatricians, cared for patients at the COE. All clinicians undergo a one month orientation on comprehensive pediatric care (including childhood TB). The clinical pediatric experience varies among COE clinicians (e.g. medical officers 0–2 years; nurses 1–3 years; pediatricians 2–4 years). In 2011, in collaboration with the National Institute for Medical Research-Mbeya Medical Research Center (NIMR-MMRC) and MZRH, the COE expanded its pediatric TB services to provide advanced diagnostics (including Xpert) and comprehensive TB treatment initiation and follow up.

### Study population and clinical procedures

Retrospective data were collected on children (<15 years of age) evaluated for presumptive TB and/or referred for TB treatment between March 2013 and December 2014. Clinicians used a combination of TB screening questions (e.g. history of known TB contact, failure to gain weight/weight loss, persistent cough, persistent fever, reduced activities or irritability) and clinical examination findings (e.g. malnutrition, lymphadenopathy, abnormal lung findings), based on national guidelines [16], to identify children with presumptive TB at the COE and in the inpatient pediatric wards at MZRH. Presumptive TB patients underwent diagnostics and evaluation at the COE, including sputa analyzed by Xpert, smear, and culture, tuberculin skin testing (TST), chest x-ray (CXR), and/or fine needle aspiration (FNA). Positive TST was defined as TST ≥5 mm. Children unable to produce sputum spontaneously completed sputum induction. The NIMR-MMRC TB lab processed sputa samples as previously described [10]. Due to low volumes of collected samples, smear and Xpert were performed routinely on the first sample received, while culture was only performed if a second sample was collected. Digital chest radiography was interpreted by the clinician using the Template Chest Radiography Review Tool [17] to assign the following grades: ‘normal,’ ‘abnormal – suspicious for TB’ (if evidence of lymphadenopathy, pleural effusion, cavities, miliary patterns, and/or airspace consolidation) and ‘abnormal – not TB’. FNA samples were collected in patients with unexplained lymphadenopathy and were sent to MZRH pathology department for histological review. All diagnostic tests were performed prior to or on the day of anti-tuberculosis treatment (ATT) initiation.

TB diagnosis was based on a combination of clinical history and findings, diagnostic results, and/or microbiological or histological data. Children with confirmed or clinically diagnosed TB were classified into one of three predefined categories of diagnostic certainty (confirmed, probable, possible TB), modeled after the internationally-accepted consensus definition of intrathoracic childhood TB [17] to also include EPTB (Table 1). Children with alternative explanations for their symptoms and clinical improvement without TB treatment were defined as “Not TB.” All patient data were reviewed throughout the 22 month study period and all children who initiated ATT were followed for the full 6 months of treatment to ensure that children diagnosed with and treated for TB had a positive treatment response and lacked an alternative, non-TB explanation of their symptoms, and that children classified as “Not TB” did not later developed TB. Treatment outcomes were based on WHO definitions [18].

**Table 1:** Diagnostic certainty categories for cases of TB disease in children^a^

Diagnostic Certainty Group	Definition of Case Categories
Confirmed tuberculosis	A child with *Mycobacterium tuberculosis* identified from a clinical specimen using any microbiologic diagnostic test available (e.g. smear, culture, or Xpert) and at least one sign or symptom suggestive of tuberculosis
Probable tuberculosis	A child with one or more of the following clinical symptoms: • Abnormal CXR consistent with TB • Cough duration greater than 2 weeks • Weight loss or failure to thrive (WHZ-score less than −2) • Fever greater than 2 weeks • Signs/symptoms of disseminated TB And one (or more) of the following: • Documented exposure to TB in the preceding 24 months • A positive clinical response to anti-tuberculosis treatment • Immunologic (TST or IGRA) evidence of *Mycobacterium tuberculosis* infection
Possible tuberculosis	A child with one or more of the following clinical symptoms: • Abnormal CXR consistent with TB • Cough greater than 2 weeks • Weight loss or failure to thrive (WHZ-score less than –2) • Fever greater than 2 weeks

^a^Adapted from Graham *et al.* [17]

### Statistical analysis

Data was extracted using a standardized data collection tool and analyzed using Stata 12.1 SE software (StataCorp2011, College Station, TX, USA). Baseline patient characteristics were summarized using descriptive statistics and compared by TB status (TB disease vs not TB disease). For patients diagnosed with TB, baseline patient characteristics were summarized using descriptive statistics and compared by confirmation status (bacteriologically confirmed vs clinically diagnosed). Age was analyzed using the Wilcoxon rank sum test, and all other categorical baseline measures were analysed using the Chi-square test to determine if these characteristics were associated with TB disease or confirmation status as appropriate. Children diagnosed with TB disease – either confirmed, possible or probable TB – were classified as “clinical TB”, which served as our primary reference standard (and incorporated both CXR and TST results). Sensitivity and specificity were calculated to assess the performance of Xpert, smear, and culture against clinical TB. Test performances were analyzed for all children and subgroups. Patients who did not have diagnostic tests performed or did not have recorded results (e.g. error/invalid) were excluded from calculations. The Kappa statistic, overall agreement, positive agreement and negative agreement were calculated to assess agreement between the three tests [19] for patients who had a positive or negative test result for all three tests.

## Results

Between March 2013 and December 2014, 455 children with presumptive TB were evaluated. Of those, 157 (34.5%) were diagnosed with TB. Baseline characteristics and diagnostic tests performed among those diagnosed with and without TB did not differ, with the exception of FNA being performed more often in those with TB compared to without [8.3% (13/157) vs 3.7% (11/298), *p* = 0.035; Table 2]. Final diagnostic certainty categories were 13.4% (21/157) bacteriologically confirmed TB, 63.1% (99/157) probable TB, and 23.6% (37/157) possible TB (Table 3). The majority of children had pulmonary TB (80.3%, 126/157). Bacteriologically confirmed TB cases were older (median age 9.4 years vs 4.6 years, *p* = 0.017) and more likely to provide sputum for analysis [100% (21/21) vs 69.1% (94/136), *p* = 0.001] compared to clinically diagnosed TB cases (Table 4).

**Table 2:** Baseline characteristics of patients referred for presumptive TB (*N* = 455)

	TB disease^a^ (*n* = 157)	Not TB disease (*n* = 298)	*p*-value
Median age in years at time of TB referral (IQR)	5.0 (1.6 – 9.5)	4.4 (1.5 – 10.0)	0.872
Male gender (%)	75 (47.8)	139 (46.6)	0.819
HIV positive (%)	84 (53.5)	163 (54.7)	0.808
Disposition at time of referral of “Inpatient” (%)	76 (48.4)	142 (47.7)	0.878
Past TB treatment (%)	7 (4.5)	9 (3.0)	0.428
Severe Malnutrition (%)^b^	50 (31.9)	119 (39.9)	0.090
Reported TB contact (%)	33 (21.0)	52 (17.5)	0.353
Diagnostic Tests Performed			
Sputum analysis (%)	115 (73.3)	205 (68.8)	0.323
Chest x-ray (%)	107 (68.2)	176 (59.1)	0.057
Tuberculin skin test (%)	143 (91.1)	264 (88.6)	0.411
Fine needle aspirate (%)	13 (8.3)	11 (3.7)	0.037

^a^TB disease includes all children meeting criteria for confirmed, possible or probable TB. ^b^Severe acute malnutrition is defined as weight-for-height Z score < 3 SD and/or presence of edema [29]

**Table 3:** Characteristics and treatment outcomes of children diagnosed with TB (*N* = 157)

Characteristic	Number	%
Treatment Initiation		
ATT initiated	155	998.7%
Median time from referral to initiation of ATT (days, IQR)	3 days	1.00–6.25 days
Diagnostic Certainty		
Confirmed TB	21	13.4%
*Confirmed pulmonary TB*	*20*	*95.2%*
*Confirmed EPTB*	*1*	*4.8%*
*Confirmed LNTB*	*0*	*0.0%*
Probable TB	99	63.1%
Possible TB	37	23.6%
Type of TB		
Pulmonary TB	126	80.3%
EPTB (excluding lymph node TB)	19	12.1%
Lymph node TB	12	7.6%
Treatment Outcomes		
Cured^a^	19	12.1%
Treatment Completed^b^	90	57.3%
Transferred Out/Not Evaluated	11	7.0%
Died	12	7.6%
Lost-to-follow up	25	15.9%

Abbreviations: *ATT*, anti-tuberculosis therapy; *EPTB*, extrapulmonary TB; *LNTB*, lymph node TB

^a^Cured = bacteriologically confirmed TB at the beginning of treatment who was smear- or culture-negative in the last month of treatment and on at least one previous occasion. ^b^Treatment Completed = clinically diagnosed or bacteriologically confirmed TB patient who completed treatment without evidence of failure, but with no record of sputum smear or culture results in the last month of treatment were negative, either because tests were not done or because no results available [18]

**Table 4:** Baseline characteristics of clinically diagnosed versus bacteriologically confirmed TB patients (*N* = 157)

	Clinically diagnosed TB (possible + probable TB) (n = 136)	Bacteriologically confirmed TB^a^ (*n* = 21)	*p*-value
Median age in years at time of TB referral (IQR)	4.6 (1.5 – 9.1)	9.4 (4.5–12.1)	0.017
Male gender (%)	68 (50.0)	14 (66.7)	0.155
HIV positive (%)	73 (53.7)	11 (52.4)	0.912
Disposition at time of referral of “Inpatient” (%)	69 (50.7)	7 (33.3)	0.137
Past TB treatment (%)	5 (3.7)	2 (9.5)	0.237
Severe Malnutrition (%)^b^	42 (30.9)	8 (38.1)	0.509
Reported TB contact (%)	28 (20.6)	5 (23.8)	0.736
Diagnostic Tests Performed			
Sputum analysis (%)	94 (69.1)	21 (100.0)	0.001
Chest x-ray (%)	91 (66.9)	16 (76.2)	0.460
Tuberculin skin test (%)	123 (90.1)	20 (95.2)	0.695
Fine needle aspirate (%)	13 (100.0)	0 (0.0)	0.218

^a^Bacteriologically confirmed TB is a TB case from whom a biological specimen is positive by smear microscopy, culture or Xpert MTB/RIF. ^b^Severe acute malnutrition is defined as weight-for-height Z score <3 SD and/or presence of edema [29]

Ninety-nine percent (155/157) of children with a TB diagnosis were initiated on ATT, with one child dying and one child lost-to-follow up (LTFU) before treatment could be initiated. Sixty-nine percent of children (109/157) showed treatment success, 7.0% (11/157) were not evaluated/transferred out, 7.6% (12/157) died and 15.9% (25/157) were LTFU (Table 3). All children followed through the completion of their treatment showed positive clinical response to TB treatment for the duration of their follow up.

**Fig. 1:**
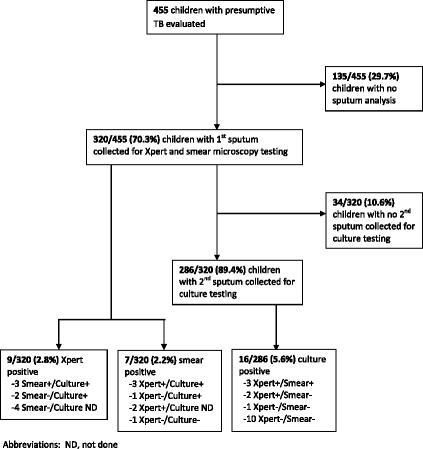
Sputum analyses performed and sputum results for children with presumptive TB

Among those producing an initial sputum (*n* = 320), 2.8% of children (9/320, 95% CI 1.3–5.3) had a positive Xpert and 2.2% of children (7/320, 95% CI 0.9–4.5) had a positive smear (Figure 1). Culture was positive in 5.6% (16/286, 95% CI 3.2–8.9) of those producing a second sputum. Xpert was positive in 7.1% (4/56, 95% CI 2.0–17.3) of the no sample for culture TB cases and none (0/85, 95% CI 0.0–4.2) of the culture negative TB cases. No rifampicin resistance was detected by Xpert.

**Fig. 2:**
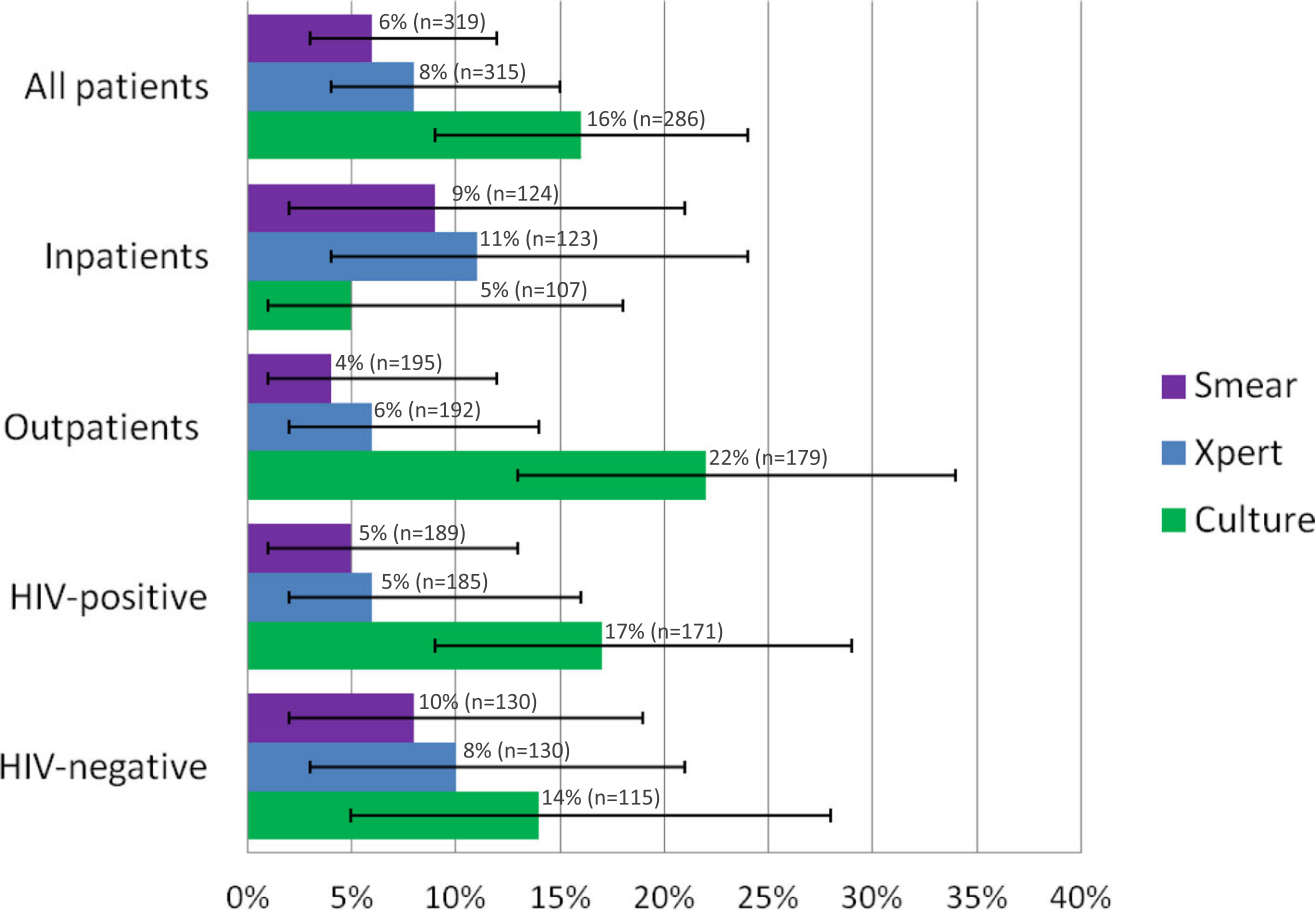
Sensitivities of smear, Xpert and culture among all patients and subgroups using “clinical TB” as the primary reference standard (with error bars representing 95% CIs)

When compared to a reference standard of clinical diagnosis, the sensitivities of culture, Xpert and smear were 16% (95% CI 9–24), 8% (95% CI 4–15), and 6% (95% CI 3–12), respectively. The sensitivity of culture, Xpert and smear did not significantly vary by inpatient versus outpatient disposition or by HIV-status (Figure 2). Specificity of culture, Xpert, and smear was high in all groups when using ‘clinical TB’ as reference standard (range of 0.99–1.00, 95% CIs 0.95–1.00). Kappa statistics for level of agreement demonstrated higher agreement for Xpert and culture compared to agreement for smear and culture in all patients and subgroups with the exception of HIV negative children (Table 5; see Additional File 1 for comparison of Xpert and clinical TB to the culture as reference standard).

**Table 5:** Agreement and discordance of culture versus Xpert and smear stratified by referral type and HIV status

	Culture versus Xpert MTB/RIF results				Culture versus smear results			
	no. with agreement/no. tested	PA (%)/NA (%)	Overall Agreement (%)	Kappa (95% CI)	no. with agreement/no. tested	Overall Agreement (%)	PA (%)/NA (%)	Kappa (95% CI)
All children	270/282	45.5/97.8	95.7	0.44 (0.18–0.70)	268/282	95.0	36.4/97.4	0.35 (0.09–0.60)
Inpatient	105/106	66.7/99.5	99.1	0.66 (0.04–1.00)	104/106	98.1	50.0/99.0	0.49 (–0.12–1.00)
Outpatient	165/176	42.1/96.7	93.8	0.40 (0.13–0.67)	164/176	93.2	33.3/96.4	0.31 (0.04–0.59)
HIV positive	160/167	53.3/97.8	95.8	0.52 (0.21–0.82)	157/167	94.0	28.6/96.9	0.27 (−0.04–0.57)
HIV negative	110/115	28.6/97.8	95.7	0.28 (−0.15–0.70)	111/115	96.5	50.0/98.2	0.49 (0.06–0.91)

PA = Positive Agreement, NA = Negative agreement

TST and CXR was performed in 89.5% (407/455) and 62.2% (283/455), respectively, of children with presumptive TB. Overall, 13.5% (55/407) of TSTs were positive and 23.3% (66/283) of CXR were resulted as ‘abnormal – suspicious for TB’ (see Additional File 2 for positivity rates of TST and CXR among subgroups). HIV positive children were more likely to have sputum analysis (76.6% vs 62.5%, p = 0.001) and less likely to have TST done (86.6% vs 92.8%, *p* = 0.03), compared to HIV negative children. There were no differences in CXR or FNA performed between the two groups.

## Discussion

Our study demonstrated that among Tanzanian children receiving care at a pediatric clinic in a resource-constrained setting, the yield of Xpert against a clinical diagnosis of TB was limited. When compared to this reference standard, the sensitivity of Xpert was 8%. Although marginally better than smear (6% sensitivity), Xpert was half as sensitive as culture (16% sensitivity). Large proportions of children with presumptive TB presented in a severely compromised state with multiple comorbidities (54.3% HIV positive, 47.9% hospitalized, 37.1% severely malnourished). This likely lowered clinicians’ thresholds for empiric TB treatment and contributed to the high numbers of clinical TB diagnoses (86.7% of TB cases). Our findings demonstrate that even with access to Xpert, TB treatment decisions rely heavily on clinical diagnosis, particularly in critically ill children.

The effect and interplay between Xpert and empiric TB treatment remains complex [20], and our findings highlight the limitations of Xpert when used in children diagnosed with culture-negative (or not performed) TB in a high burden setting. Our estimates of Xpert sensitivity are consistent with previous research comparing the performance of Xpert to clinical diagnosis in culture-negative pediatric cases [5, 21, 22], as well as an earlier prospective study in our same study setting [10] that demonstrated 8.5% sensitivity of Xpert in children clinically diagnosed with TB. While that study evaluated three sputa samples for every patient, our programmatic approach allowed a maximum of one sputum sample for culture. The use of multiple sputum samples improves Xpert test performance [10, 23], however this approach is not feasible in our, nor in many other, resource-constrained settings. A thoughtful comparison of these differences further highlights the reality and challenges of TB diagnostics, care and treatment of children presenting in the clinical care arena rather than a research setting.

Emerging evidence suggests under-diagnosis of TB may be common in children with severe acute malnutrition (SAM) and/or HIV and their mortality is high [7, 24, 25]. We were able to evaluate TB diseased children who presented with a wide spectrum of disease severity and co-morbidities – including HIV co-infection, SAM, and inpatient disposition – and were unable to show a difference in performance of Xpert in children based on their HIV, malnutrition, or inpatient status. We postulate that in these children clinicians have lower thresholds for empiric TB treatment, and their TB infection may have progressed more rapidly to severe disease, despite lower, undetectable mycobacterial loads.

The impact of any new diagnostic test – such as Xpert – depends greatly on how it is implemented outside of research settings and on the context in which it is implemented [26]. Two recent randomized controlled trials evaluating the impact of Xpert in adults in high TB burden settings showed that despite increased sensitivity and decreased turnaround time, its use did not translate into patient important outcomes such as reduced morbidity or mortality [27, 28]. Xpert is particularly useful when positive in pediatric cases where a clinical diagnosis is unclear. However, a negative Xpert result cannot exclude the possibility of TB disease, especially in children living in areas of high TB burden. In our setting, the majority of childhood TB cases were still diagnosed clinically, demonstrating how, even though Xpert is a valuable additional test in children, it is not a stand-alone replacement to culture and clinical diagnosis. Clinicians must be made aware of these limitations and resources should be utilized to strengthen all components of the diagnostic algorithm in concert.

Our study was limited by its retrospective approach assessing TB care at a single site. Despite clinical practice following national guidelines, there was variability in the diagnostic tests performed per patient, reflecting the reality of clinical practice in TB high burden, resource constrained settings. This variability provided unique and realistic insights regarding the incorporation of new diagnostics into clinical care. Our site received many referrals, contributing to potential referral bias that lowered clinicians’ thresholds to treat empirically. High levels of empiric treatment may limit the performance of Xpert against clinical practice. Although there were few bacteriologically confirmed cases, our study benefited from long follow up of at least six months of all patients to ensure that clinically diagnosed TB cases improved on ATT and ‘not TB’ cases improved without ATT. Our study did not specifically look at impact of Xpert on clinician behavior or favorable patient outcomes, but provided the basic foundation for such future research. Overall, these findings emphasize the importance of evaluating new diagnostics in children against clinical diagnosis as reference standards.

## Conclusion

Despite access to Xpert technology, the majority of childhood TB cases presenting to a pediatric referral centre in a resource limited setting were diagnosed clinically, thus limiting the contribution of Xpert to clinical decision making under our programmatic conditions. Recognizing the paucibacillary nature of childhood TB and high risk of rapid disease progression in children, new diagnostics such as Xpert serve as important adjunctive tests but will not obviate the need for astute clinicians and comprehensive diagnostic algorithms. Care should be taken to carefully balance allocation of scarce resources between new diagnostic technologies (e.g. Xpert) and educational efforts to foster traditional components of the diagnostic algorithm (e.g. history, physical exam, radiography, TST) for childhood TB in resource limited settings.

## Additional files

Additional file 1: Performance outcomes of clinical TB diagnosis and Xpert MTB/RIF using culture as reference standard for patients <15 years old and referred for presumptive TB. The data in additional file 1 shows the sensitivity, specificity, PPV and NPV of clinical TB and Xpert MTB/RIF compared to culture as the reference standard for all patients as well as the subgroups of inpatients, outpatients, HIV positive and HIV negative. (DOCX 12 kb)
Additional file 2: Tuberculin Skin Test (TST) and chest x-ray positivity rates among children with presumptive TB. The data in additional file 2 shows the positivity rates of TST and CXR for subgroups of presumptive TB, TB disease, HIV positive, HIV negative, inpatients and outpatients. (DOCX 11 kb)
