# Supplementary material for: Why being an expert – despite xpert –remains crucial for children in high TB burden settings
Source: BMC Infect Dis. 2017 Feb 6;17:123. doi: 10.1186/s12879-017-2236-9 (PMC5294844; doi:10.1186/s12879-017-2236-9)
Supplement: Additional file 1: — Performance outcomes of clinical TB diagnosis and Xpert MTB/RIF using culture as reference standard for patients <15 years old and referred for presumptive TB. The data in additional file 1 shows the sensitivity, specificity, PPV and NPV of clinical TB and Xpert MTB/RIF compared to culture as the reference standard for all patients as well as the subgroups of inpatients, outpatients, HIV positive and HIV negative. (DOCX 12 kb) [file 12879_2017_2236_MOESM1_ESM.docx]

|  |  |  |
| --- | --- | --- |
|  | Clinical TB diagnosis | Xpert MTB/RIF |
| *Sensitivity* |  |  |
| All patients (95% CI) | 0.94 (0.71-1.00) [n=286] | 0.29 (0.10-0.56) [n=282] |
| Inpatients (95% CI) | 1.00 (0.16-1.00) [n=107] | 0.50 (0.01-0.99) [n=106] |
| Outpatients (95% CI) | 0.93 (0.68-1.00) [n=179] | 0.27 (0.08-0.55) [n=176] |
| HIV-infected (95% CI) | 0.91 (0.59-1.00) [n=171] | 0.36 (0.11-0.69) [n=167] |
| HIV-uninfected (95% CI) | 1.00 (0.54-1.00) [n=115] | 0.17 (0.42-0.64) [n=115] |
|  |  |  |
| *Specificity* |  |  |
| All patients (95% CI) | 0.68 (0.63-0.74) [n=286] | 1.00 (0.99-1.00) [n=282] |
| Inpatients (95% CI) | 0.66 (0.56-0.75) [n=107] | 1.00 (0.97-1.00) [n=106] |
| Outpatients (95% CI) | 0.70 (0.63-0.77) [n=179] | 1.00 (0.98-1.00) [n=176] |
| HIV-infected (95% CI) | 0.70 (0.62-0.77) [n=171] | 1.00 (0.98-1.00) [n=167] |
| HIV-uninfected (95% CI) | 0.66 (0.56-0.75) [n=115] | 1.00 (0.97-1.00) [n=115] |
|  |  |  |
| *PPV* |  |  |
| All patients (95% CI) | 0.16 (0.09-0.24) [n=286] | 1.00 (0.66-1.00) [n=282] |
| Inpatients (95% CI) | 0.05 (0.01-0.18) [n=107] | 1.00 (0.03-1.00) [n=106] |
| Outpatients (95% CI) | 0.22 (0.13-0.35) [n=179] | 1.00 (0.40-1.00) [n=176] |
| HIV-infected (95% CI) | 0.17 (0.09-0.29) [n=171] | 1.00 (0.40-1.00) [n=167] |
| HIV-uninfected (95% CI) | 0.14 (0.05-0.28) [n=115] | 1.00 (0.03-1.00) [n=115] |
|  |  |  |
| *NPV* |  |  |
| All patients (95% CI) | 1.00 (0.97-1.00) [n=286] | 0.96 (0.93-0.98) [n=282] |
| Inpatients (95% CI) | 1.00 (0.95-1.00) [n=107] | 0.99 (0.95-1.00) [n=106] |
| Outpatients (95% CI) | 0.99 (0.95-1.00) [n=179] | 0.94 (0.89-0.97) [n=176] |
| HIV-infected (95% CI) | 0.99 (0.95-1.00) [n=171] | 0.96 (0.91-0.98) [n=167] |
| HIV-uninfected (95% CI) | 1.00 (0.95-1.00) [n=115] | 0.96 (0.90-0.99) [n=115] |
|  |  |  |
