# Supplementary material for: Why being an expert – despite xpert –remains crucial for children in high TB burden settings
Source: BMC Infect Dis. 2017 Feb 6;17:123. doi: 10.1186/s12879-017-2236-9 (PMC5294844; doi:10.1186/s12879-017-2236-9)
Supplement: Additional file 2: — Tuberculin Skin Test (TST) and chest x-ray positivity rates among children with presumptive TB. The data in additional file 2 shows the positivity rates of TST and CXR for subgroups of presumptive TB, TB disease, HIV positive, HIV negative, inpatients and outpatients. (DOCX 11 kb) [file 12879_2017_2236_MOESM2_ESM.docx]

| Patient group  (n completing the diagnostic test) | Positivity Rate* |
| --- | --- |
| TST |  |
| Presumptive TB (n=407) | 13.5% (55/407) |
| TB disease^†^ (n=143) | 28.7% (41/143) |
| HIV positive (n=214) | 9.3% (20/214) |
| HIV negative (n=193) | 18.1% (35/193) |
| Inpatients (n=197) | 9.6% (19/197) |
| Outpatients (n=210) | 17.1% (36/210) |
| Chest x-ray |  |
| Presumptive TB (n=283) | 23.3% (66/283) |
| TB disease^†^ (n=107) | 57.0% (61/107) |
| HIV positive (n=155) | 25.8% (40/155) |
| HIV negative (n=128) | 20.3% (26/128) |
| Inpatients (n=122) | 14.8% (18/122) |
| Outpatients (n=161) | 29.8% (48/161) |

*Positive test result for TST defined as TST ≥5mm; positive test result for chest x-ray defined as x-ray read as ‘abnormal – suspicious for TB’ (i.e. evidence of lymphadenopathy, pleural effusion, cavities, miliary patterns, and/or airspace consolidation); † TB disease includes probable TB and possible TB cases, both of which incorporate TST and CXR results into their case definitions
